# Restoring Dystrophin Expression in Duchenne Muscular Dystrophy: Current Status of Therapeutic Approaches

**DOI:** 10.3390/jpm9010001

**Published:** 2019-01-07

**Authors:** Yuko Shimizu-Motohashi, Hirofumi Komaki, Norio Motohashi, Shin’ichi Takeda, Toshifumi Yokota, Yoshitsugu Aoki

**Affiliations:** 1 Department of Child Neurology, National Center Hospital, National Center of Neurology and Psychiatry, 4-1-1 Ogawahigashi-cho, Kodaira, Tokyo 187-8551, Japan; ymotohashi@ncnp.go.jp; 2 Department of Molecular Therapy, National Institute of Neuroscience, National Center of Neurology and Psychiatry, 4-1-1 Ogawahigashi-cho, Kodaira, Tokyo 187-8502, Japan; takeda@ncnp.go.jp; 3 Translational Medical Center, National Center of Neurology and Psychiatry, 4-1-1 Ogawahigashi-cho, Kodaira, Tokyo 187-8502, Japan; komakih@ncnp.go.jp; 4 Department of Geriatric Medicine, Tokyo Metropolitan Institute of Gerontology, Itabashi, Tokyo, 173-0015, Japan; nmotoha@tmig.or.jp; 5 Department of Medical Genetics, Faculty of Medicine and Dentistry, University of Alberta. 831 Medical Sciences Building, 8613-114 St., Edmonton, AB T6G 2H7, Canada; toshifum@ualberta.ca

**Keywords:** read-through, exon skipping, vector-mediated gene therapy, cell therapy

## Abstract

Duchenne muscular dystrophy (DMD), a rare genetic disorder characterized by progressive muscle weakness, is caused by the absence or a decreased amount of the muscle cytoskeletal protein dystrophin. Currently, several therapeutic approaches to cure DMD are being investigated, which can be categorized into two groups: therapies that aim to restore dystrophin expression, and those that aim to compensate for the lack of dystrophin. Therapies that restore dystrophin expression include read-through therapy, exon skipping, vector-mediated gene therapy, and cell therapy. Of these approaches, the most advanced are the read-through and exon skipping therapies. In 2014, ataluren, a drug that can promote ribosomal read-through of mRNA containing a premature stop codon, was conditionally approved in Europe. In 2016, eteplirsen, a morpholino-based chemical capable of skipping exon 51 in premature mRNA, received conditional approval in the USA. Clinical trials on vector-mediated gene therapy carrying micro- and mini- dystrophin are underway. More innovative therapeutic approaches include CRISPR/Cas9-based genome editing and stem cell-based cell therapies. Here we review the current status of therapeutic approaches for DMD, focusing on therapeutic approaches that can restore dystrophin.

## 1. Introduction

Duchenne muscular dystrophy (DMD) is a muscular disorder caused by the absence or reduction of the muscle cytoskeletal protein dystrophin. The causative gene, *DMD*, is located on Xp21, and dystrophin, which is its protein product, links the sarcomeric structure to the extracellular matrix [1] to protect the sarcolemma from contraction-induced injury [2]. Male individuals are primarily affected because of the nature of X-linked recessive inheritance, although female carriers can occasionally develop symptoms when they have skewed X inactivation or chromosomal conditions with an abnormality in one of the X chromosomes [3].

Patients with DMD manifest progressive muscular weakness and eventually develop respiratory and cardiac failure, critical factors that determine their survival. The first sign of muscle weakness is detected at the age of 2–5 years, during which the symptoms slowly progress and render the patient non-ambulatory. Respiratory and cardiac functions are affected and worsen as the disease stage advances, and patients can currently live up to their 30s with a multidisciplinary treatment approach [4].

Data retrieved from a global database revealed that in DMD, 80% of all mutations were large mutations involving at least one exon or more [5]. Of the large mutations, 86% were deletions and 14% were duplications [5]. The remaining 20% of all mutations were small mutations, which affected segments smaller than one exon, and half were nonsense mutations [5].

Patients with a mutation in *DMD* can present variability in their symptoms, ranging from the most severe DMD to the milder form, which is known as Becker muscular dystrophy. The severity difference is attributed to the reading frame of the gene mutation. In DMD, the mutation is “out-of-frame”, which indicates that the translational reading frame is disrupted, leading to the loss of dystrophin [6]. When a mutation is “in-frame”, i.e., a short but functional dystrophin can be produced, individuals may manifest a less severe phenotype [6]. However, the severity of symptoms in some patients cannot be explained by this “reading frame hypothesis”, and the underlying reason for these exceptions remains unclear. In patients with nonsense mutations, stop codons are generated leading to premature translational termination [7].

To date, several therapeutic approaches to treat DMD have been investigated that can be divided into two groups: therapies that aim to restore dystrophin expression, and those that aim to compensate for the lack of dystrophin [8]. The former group includes read-through therapy, exon skipping therapy, vector-mediated gene therapy, and cell therapy. The latter group includes anti-inflammatory, anti-fibrotic, antioxidants, myostatin pathway inhibition, neuronal nitric oxide synthase pathway enhancement, and utrophin upregulation therapies. Of these approaches, read-through and exon skipping therapies have taken the development lead. In this article, we review the current status of therapeutic approaches (Table 1) that can restore dystrophin for patients with DMD.

## 2. Read-Through Therapy

Read-through therapy is an approach applicable to patients with nonsense mutations. Approximately 10% of all patients with DMD carry nonsense mutations, and these patients may benefit from this therapy [5]. The nonsense mutation results in a premature stop codon in the dystrophin mRNA, leading to the translation of a truncated, nonfunctional protein [9]. Additionally, the mRNA generated from a nonsense mutation is destabilized by nonsense-mediated mRNA decay (NMD); thus, the inactivation of any factor related to this pathway may stabilize the transcript [7].

It has been shown that aminoglycoside antibiotics can read through premature nonsense codons [10,11]. Results of in vitro and in vivo studies have shown that gentamycin administration to *mdx* mice, an animal model of DMD harboring a nonsense mutation in exon 23 of *Dmd* [12], can restore dystrophin expression and resistance to contraction-induced muscle injury [13]. A clinical trial involving patients with nonsense mutations in DMD receiving an intravenous injection of gentamycin once daily for 2 weeks demonstrated safety; however, full-length dystrophin could not be detected in pre- and post-treatment muscle biopsies [14]. Another study reported that patients who received gentamycin for 6 months exhibited significantly increased dystrophin levels in their post-treatment muscle tissues [15]. A drawback of gentamycin is the renal and otic toxicities of the drug [8].

Ataluren (3-[5-(2-fluorophenyl)-[1,2,4]oxa-diazol-3-yl]-benzoic acid; C_15_H_9_FN_2_O_3_), a compound formerly called PTC124, can promote nonsense suppression with less toxicity. Ataluren was identified via high-throughput screening [7] and developed by PTC Therapeutics. In phase 2a, an open-label study that assessed patients with DMD reported increased dystrophin protein levels in a subset of participants [16]. The phase 2b, double-blind, randomized placebo-controlled trial (RCT) demonstrated a non-significant but favorable result in the 6-min walk test (6MWT) in the ataluren treated group [17]. The data from the phase 3, double-blind, randomized placebo-controlled trial assessing 230 patients from 18 countries did not demonstrate a significant difference in the 6MWT distance between the ataluren treated and the placebo-treated groups; only in a prespecified subgroup with a baseline 6MWT distance of 300–399 m was there a significant difference in 6MWT distance compared with the placebo-treated group [9]. The recent preliminary patient registry data indicate a longer ambulatory period in ataluren-treated patients compared with published natural history [18].

Because the phase 2 clinical trial results were not satisfactory in terms of meeting the primary endpoint, ataluren was first rejected for approval by the European Medicines Agency (EMA) [19]. However, the conclusions drawn from further analysis suggested that ataluren was effective in terms of slowing down the disease course, leading EMA to grant ataluren a conditional marketing authorization in 2014 [20]. Currently, the drug is available in Europe for treating patients who are aged ≥2 years with nonsense *DMD* mutations [20], whereas ataluren is still an investigational drug in the United States [21].

## 3. Exon Skipping

Exon skipping is a therapeutic approach that can correct the disrupted reading frame in patients with DMD. The reading frame is corrected by skipping a targeted exon with pre-designed antisense oligonucleotides (ASOs), which restores the disrupted reading frame of *DMD* by modulating the dystrophin pre-mRNA splicing process. Through this modulation, a shorter but functional dystrophin is generated, which converts the severe DMD phenotype to a milder BMD phenotype.

Theoretically, exon skipping is applicable to approximately 80% of total *DMD* mutations [22]. These mutations include deletions, duplications, and small mutations (deletions, insertions, splice site mutations, and point mutations). A mutation hotspot (where mutations tend to accumulate) is present between exons 45 to 55 in *DMD* [5]. Of all *DMD* mutations, exon 51 skipping is applicable to 14% of patients, representing the largest DMD population who may benefit from single exon skipping [5]. Therefore, drug targeting to skip exon 51 was first investigated.

Phosphorodiamidate morpholino oligomer (PMO) and 2’-*O*-methyl-phosphorothioate oligonucleotide (2’*O*MePS) are oligonucleotide drugs known to possess high stability, high efficacy, and low toxicity [23]. In terms of chemical difference, PMO is neutrally charged, whereas 2’*O*MePS is negatively charged. PMO neutrality is considered to reduce the risk of side effects in terms of reducing off-target effects and immune responses [24]. The two major ASOs developed to skip exon 51 were eteplirsen (developed by Sarepta Therapeutics) using PMO and drisapersen (developed by Prosensa) using 2’*O*MePS.

A double-blind, randomized, placebo-controlled study including 12 patients has demonstrated the safety of eteplirsen but could not demonstrate clear clinical efficacy [25]. After overcoming the controversial advisory committee [26], eteplirsen received conditional approval from the US Food and Drug Administration (FDA) for DMD treatment in 2016 [27]. However, the FDA has mandated post-marketing clinical trials to prove the efficacy of eteplirsen, with results to be reported by 2021 [26]. A more recent report has indicated that after 3 years of follow up, eteplirsen-treated patients exhibited a slower rate of ambulation decline compared with the historical control data [28].

Clinical trials of drisapersen could not demonstrate efficacy, and furthermore, significant renal and injection site reaction were observed in the drisapersen-treated group compared with the placebo-treated group [24]. Lack of apparent efficacy and safety concerns have led the FDA to not approve drisapersen in 2015 [26]. To note, combined data of double-blind RCTs evaluating the efficacy of drisapersen have revealed that when drisapersen was used at a dose of 6 mg/kg weekly injection, the treated group had significantly favorable outcomes in the 6MWT [24].

To date, the exon skipping therapies have expanded with other exons targeted by PMO, among which the exon 53 skipping drug is in the most advanced stage [29,30]. Recently, a report on a phase 1, open-label, dose escalation clinical trial studying NS-065/NCNP-01 (Nippon Shinyaku Co. Ltd.) was published, demonstrating safety of the drug and its ability to induce exon 53 skipped mRNA in all patients [30]. A phase 1/2, double-blind, placebo-controlled study with extension to an open-label, long-term study of SRP-5053 (Sarepta Therapeutics) demonstrated the induction of exon 53 skipped mRNA [29]. Furthermore, clinical trials to skip exon 45 by PMO are currently ongoing [31].

The pharmacodynamics of cellular uptake and renal clearance from circulation of PMO indicate a requirement of large and repeated doses for therapeutic application [32]. Additionally, PMO shows only a limited exon-skipping effect in the cardiac muscle in animal models [32]; however, the efficacy of each compound is expected to vary greatly, depending on its chemical, sequence optimization, and dosage.

To improve the intracellular delivery of PMO, a peptide-conjugated morpholino oligomer (PPMO) has been considered as a promising compound. Compared with PMO, PPMO is less affected by serum and more stable in the blood [33] and is more efficiently delivered to cells [32]. If PPMO overcomes the issues observed in PMO and higher levels of dystrophin are produced, more efficient dosing for patients may be anticipated following this modification. Safety should be considered for PPMO because of the cationic nature of the peptide predicting a higher toxicity than for PMO [32]. Moreover, when administered to animals at a high dose, PPMO induced lethargy, weight loss, and renal toxicity [34]. A clinical trial to treat patients amenable to exon 51 skipping with PPMO (NCT03375255) is currently underway.

Another promising exon skipping compound developed by WAVE Life Sciences Ltd. (Cambridge, MA, USA) is stereopure ASO [35,36]. The conventional method synthesizes ASOs with randomly oriented atoms at each linkage of nucleotides, adopting either Sp or Rp position [35]. Therefore, because of the pharmacologic properties of traditional ASOs including more than 500,000 permutations within one dose, unstable therapeutic effects and off-target effects are obtained [35]. The idea of stereopure ASO is to construct a more homogeneous set of molecules within the dosage, anticipating increased safety and effectiveness [35]. Stereopure ASO to skip exon 51 is currently being tested in a clinical trial (NCT03508947).

Other promising ASOs include an antisense oligonucleotide consisting of ethylene-bridged nucleic acids, DS-5141b, (Daiichi-Sankyo Co., Ltd., Tokyo, Japan) to skip exon 45, which recently was announced to be safe and has shown mRNA exon 45 skipping in all treated patients in a phase 1/2 trial [37].

## 4. Vector-Mediated Gene Therapy

Delivery of normal *DMD* to replace the affected gene with a functional gene has been conceptually perceived as an attractive therapeutic approach. Because of the enormous size of *DMD* (2.4 Mb) and its cDNA (14 kb) and the broad distribution of affected organs, this therapeutic approach appears challenging. The identification of patients with a mild phenotype who carry large deletions [6,38] led to a suggestion that delivery of specific domains of dystrophin could be functional [39]. Further reports have described the N-terminal or cysteine-rich domain as the crucial domain for dystrophin to be functional [40,41,42].

Vector-mediated gene therapy for DMD consists of delivering functional domains of dystrophin via viral or non-viral vectors to restore dystrophin. Of the viral vectors investigated, adeno-associated virus (AAV) vectors are in the most advanced stage of drug development. AAVs possess defective replication and low immunogenicity, with long-term transgene expression over many years in nondividing cells and no known pathogenicity [43]. The maximum packaging capacity of an AAV vector is 5 kb [44], and the discovery of micro-dystrophin made delivery via AAV vector possible as shown by the effectiveness in muscle in *mdx* mice [43,45]. Micro-dystrophins are functional, smaller cDNA clones, and more than 30 different configurations of synthetic micro-dystrophins have been reported to date [44].

The systemic delivery of AAV vector-mediated gene transfer became available with AAV in animal models, first in rodents [46,47] and then in canines [48]. These studies have demonstrated safety and amelioration of muscle pathology in animals, and furthermore, the transduction of *DMD* was observed also in cardiac muscle [46,47,48], which is important because cardiomyopathy is the major cause of death in patients with DMD.

In 2006, the first AAV vector-mediated micro-dystrophin therapy was initiated, and the minigene cassette packaged in AAV 2.5 was injected into the biceps of six patients aged 5–11 years old [49,50]. The muscle biopsy was performed at 43 or 90 days after the injection, revealing limited expression of the transduced dystrophin in two of six patients and none in the four other patients [50]. However, in patients with no detectable micro-dystrophin, two had elevated T cell responses to dystrophin epitopes from endogenous and/or transgene products after vector delivery, indicative of transgene expression even when the functional protein was undetectable [49,50], and a third patient exhibited T cell response to AAV capsid [44,49,50]. The unfavorable result in this study suggested several possibilities, such as immunity to new antigenic epitopes in transduced micro-dystrophin, immunity to preexisting antigenic epitopes in revertant dystrophin, and immunity to viral capsid [44]. Recent research has shown promising results of treatment with an engineered micro-dystrophin DNA plasmid vaccine dampening the immune response to both dystrophin and AAV capsid in an animal model [51].

Among clinical trials conducted in the USA, two were initiated in 2017 (NCT03368742, NCT03375164) and one in 2018 (NCT03362502). All studies are open-label, and the primary outcomes are safety, with the NCT03368742 study assessing micro-dystrophin expression in biopsy as primary outcome and the other two as a secondary outcome [44]. Patients with any mutation are included in the NCT03368742 and the NCT03362502 studies, whereas the NCT03375164 study targets patients with frameshift or nonsense mutations within exons 18–58 [44]. The serotypes of AAV are AAV-9 in the NCT03368742 and NCT03362502 studies and AAV-rh74 in the NCT03375164 study [44]. Notably, the NCT03375164 study includes children as young as 3 months old. The studies are estimated to end in 2021 (NCT03368742, NCT03375164) and 2024 (NCT03362502) [44].

## 5. Cell Therapy

Cell therapy involves transplanting cells that are capable of producing functional dystrophin into patients with DMD. The cells can either be genetically unmodified cells from healthy donors or autologous genetically corrected cells [8]. The pros and cons of these cell sources would be that unmodified cells from healthy donors are mutation-free but have a risk of immune reaction, whereas autologous genetically corrected cells have a lower risk of immune reaction but require manipulation of the gene prior to the therapy [52]. Several cell types are considered to be candidates for therapy, including satellite cells, myoblasts [53,54,55], CD133+ stem cells [56,57], mesoangioblasts [58,59,60], pericytes [61], mesenchymal stem cells [62], embryonic stem cells [63], and induced pluripotent stem (iPS) cells [63,64]. So far, the clinical trials of myoblasts [53], CD133+ stem cell [56] and mesoangioblast [60] used cells from healthy donors, and in preclinical studies, autologous cell transplantation after genetic correction of DMD mutation has been tested in animal models [65,66,67].

Transplantation of normal myogenic cells into dystrophin-deficient muscle was an attractive therapeutic approach. Satellite cells expressing Pax7, located underneath the basal lamina of muscle fibers, are considered to be stem cells for skeletal muscle [68]. Satellite cells transform into myoblasts upon injury to generate new muscle fibers [69]. Because of their high ability to generate muscle fibers, satellite cells and myoblasts are considered good candidates for cell therapy in patients with DMD [8]. However, myoblast transplantation has several limitations, including immune rejection, poor cellular survival rates of the injected cells [70]. Additionally, satellite cells lack the ability to cross the endothelium, and have limited ability to migrate [61]. Therefore, systemic intravascular delivery of this cell source possesses technical challenges. Clinical trials were conducted to treat patients with DMD by intramuscular injection of normal human satellite cells or myoblasts and had shown some expression of donor-derived dystrophin [53,54,55], but in a study that measured functional outcome, functional amelioration could not be demonstrated [53]. A clinical trial transplanting myoblasts is ongoing, and is expected to be completed in 2019 (NCT02196467).

CD133+ stem cells are blood- [57] and muscle-derived [65] stem cells that are able to differentiate into muscle, hematopoietic, and endothelial cells when exposed to the appropriate cytokines. When injected into dystrophin-deficient mdx mice, the CD133+ cells could migrate toward myofibers, contributing to the muscle fiber regeneration [57,65]. In addition, human muscle-derived CD133+ cells could contribute to the re-constitution of satellite cells after intramuscular transplantation [71]. A clinical trial involving transplantation of muscle-derived CD133+ stem cells demonstrated safety of this cell source [56].

Mesoangioblasts are vessel-associated multipotent stem cells participating in postembryonic development of the mesoderm [58]. A previous report demonstrated that mesoangioblasts isolated from the embryonic dorsal aorta can differentiate into muscle fibers [58]. When normal mesoangioblasts were delivered to the canine model of DMD via arteries, the animal exhibited recovery of dystrophin expression and muscle function [59]. A clinical trial of intra-arterial mesoangioblast transplantation in patients with DMD demonstrated relative safety, but one out of five patients developed thalamic stroke of unclear relevance to the therapy [60]. In this study, the effect of mesoangioblast transplantation on muscle function was inconclusive [60].

Pericytes are the contractile connective tissue cells surrounding the microvasculature in adult tissue, and are thought to be developmentally derived from mesoangioblasts [61]. These cells have the ability to differentiate into adipocytes, chondrocytes or myogenic cells [61,72]. Human pericytes from DMD patients, where a human mini-dystrophin was transduced by lentiviral vector, were transplanted into immuno-deficient mdx (scid-mdx) mice gave rise to myofibers expressing human dystrophin [61]. Since CD133+ cells, mesoangioblasts and pericytes have the myogenic potential with the ability to penetrate the blood vessel wall, these cells are considered as promising candidate cell sources to treat patients with DMD.

The transplantation of human iPS-derived myogenic cells into *mdx* mice produced human-derived dystrophin-positive muscle fibers and improved muscle strength [63]. Furthermore, frameshifting, exon knock-in, or exon skipping in patient-derived human iPS cells using CRISPR/Cas9 technology was reported, which may permit ex-vivo gene correction followed by autologous cell transplantation in patients with DMD [64,73].

## 6. Challenges and Limitations in Therapies to Restore Dystrophin

Evidence of recent therapeutic approaches to restore dystrophin in patients with DMD has been accumulating, with several data demonstrating positive effects both in animal models and in humans. The discovery of read-through and exon skipping has ushered in a new era in DMD therapy; although marketed in limited countries, ataluren and eteplirsen are now available to treat patients with DMD. The efficacies of these therapeutic approaches; however, are still ambiguous.

Multiple issues hinder the generation of evidence for therapeutic approaches in DMD. From the clinical point of view, one major issue is the rarity of DMD, with only a small number of patients capable of participating in a study to assess the efficacy of novel drugs. Well-designed studies to prove drug efficacy, such as RCTs with sufficient statistical power, are extremely difficult to enroll. Another problem with conducting clinical trials in DMD is that there is no specific set of recommended or required outcome measures [74,75], and outcomes that can adequately reflect the subtle, but important change in the clinical course of DMD are unknown [24]. Study designs that incorporate the results of small, underpowered study results into a prospectively planned meta-analysis and development and utilization of surrogate markers may be helpful to overcome these problems [76].

From the preclinical point of view, several issues need to be addressed. Notably, for exon skipping, improvement in the target-tissue uptake efficiency of ASOs should be pursued. To generate ASOs with enhanced cellular uptake, alterations of chemical backbones, including PPMOs, have been studied. Furthermore, facilitation of enhanced PPMOs uptake via self-assembly into nanoparticles has been reported [77]. Other issues include whether the sequence of currently available ASOs is optimal since the efficacy of exon skipping at different target positions can vary more than 20-fold [78].

For vector-mediated gene therapy, improvement in AAV capsid, maximization of micro-dystrophin potency, and minimization of the immunological risk are still needed [44]. An important issue for this therapeutic approach is whether micro-dystrophin can treat DMD in humans with regard to the length, the number of repeats, and the presence of the central hinge [44]. Patients who have lost ≥50% of dystrophin due to large in-frame deletion often exhibit a severe phenotype [44]. Additionally, there may be a length threshold in dystrophin [44,79], i.e., a protein that is small or truncated in length may not be protective. Further preclinical studies aim to deliver larger or full-length dystrophin via human artificial chromosome [80], lentiviral vectors [81], or foamy viral vector [82]. Moreover, the durability of the therapy is a cause for concern, but systemic administration of micro-dystrophin in the canine model resulted in significant and sustained levels of micro-dystrophin in skeletal muscles and reduced disease symptoms for at least 2 years [83]. Because of the degenerative nature of the disease, it is possible that patients with DMD have to receive repeated doses; however, the antibody response from initial exposure to AAV therapy represents a barrier for re-administration [44]. Studies to overcome the immune response are currently underway [44].

The limitations of cell therapy in treating patients with DMD include the limited number of cells available, low survival rate and migration ability of injected cells, potential tumor formation due to mis-differentiation, and immune response to donor cells [52]. Furthermore, myogenic potentials, transplantation efficiency, and ways to systemic delivery of the cells are needed to be addressed for the clinical use.

## 7. Conclusions

To date, read-through and exon skipping drugs have received conditional approval. Vector-mediated gene therapy is awaited for clinical usage, and approval of this therapy enables to cover the broader number of patients who would not benefit from read-through or exon skipping. Owing to minor changes in dystrophin production or nonsignificant functional improvements reported to date, the clinical effect could be subtle and difficult to identify during clinical trials, which take place for a relatively short period of time. While necessary in such cases, studies involving a control population are not feasible in DMD. Optimization of study designs, or structures of the drug may help to evaluate drug efficacy in the future. Furthermore, analyzing real-world data may provide information on the true efficacy of innovative drugs, such as improvement in survival, which can only be assessed after long-term usage of the drug.

## Figures and Tables

**Table 1:** Ongoing clinical trials ^1^ and approval status of therapeutic approaches to restore dystrophin in Duchenne muscular dystrophy.

Drug/Compound	Description	Company/Institute	Clinical Trial Number	Status
Ataluren	Read-through	PTC Therapeutics		Conditional approval in Europe
			NCT01247207	Phase 3
			NCT01557400	Phase 3
			NCT02090959	Phase 3
			NCT03179631	Phase 3
Eteplirsen	Exon skip PMO targeting exon 51	Sarepta Therapeutics		Conditional approval in the USA
			NCT02255552	Phase 3
			NCT02420379	Phase 2
			NCT03218995	Phase 2
SRP-4053 (Golodirsen)	Exon skip PMO targeting exon 53	Sarepta Therapeutics	NCT02310906	Phase 1/2
			NCT02500381	Phase 3
			NCT03532542	Phase 3
SRP-4045 (Casimersen)	Exon skip PMO targeting exon 45	Sarepta Therapeutics	NCT02500381	Phase 3
			NCT02530905	Phase 1
			NCT03532542	Phase 3
SRP-5051	Exon skip PPMO targeting exon 51	Sarepta Therapeutics	NCT03375255	Phase 1
			NCT03675126	Phase 1/2
NS-065/NCNP-01	Exon skip PMO targeting exon 53	NS Pharma, Inc., Nippon Shinyaku Co., Ltd., Cooperative International Neuromuscular Research Group, Therapeutic Research in Neuromuscular Disorders Solutions (TRiNDS)	NCT03167255	Phase 2
DS-5141b	ENA Exon skip targeting exon 45	Daiichi Sankyo Co., Ltd., Orphan Disease Treatment Institute Co., Ltd., Daiichi Sankyo, Inc.	NCT02667483	Phase 1/2
WVE-210201	Steropure ASO Exon skip targeting exon 51	Wave Life Sciences Ltd.	NCT03508947	Phase 1
rAAVrh74.MCK.GALGT2	Micro-dystrophin Gene Transfer	Nationwide Children’s Hospital (USA)	NCT03333590	Phase 1/2
PF-06939926	Mini-dystrophin gene transfer	Pfizer	NCT03362502	Phase 1
SGT-001	Micro-dystrophin gene transfer	Solid Biosciences, LLC	NCT03368742	Phase 1/2
rAAVrh74.MHCK7.micro-dystrophin	Micro-dystrophin gene transfer	Nationwide Children’s Hospital, Washington University School of Medicine (USA)	NCT03375164	Phase 1/2
Myoblasts	Cell therapy	CHU de Quebec-Universite Laval (Canada)	NCT02196467	Phase 1/2
Umbilical cord mesenchymal stem cells	Cell therapy	Allergy and Asthma Consultants (USA), Aidan Foundation	NCT02235844	Phase 1
Bone marrow-derived autologous stem cells	Cell therapy	Stem Cells Arabia	NCT03067831	Phase 1/2

^1^ Clinical trials that are recruiting, not yet recruiting, enrolling by invitation, or active but not recruiting. https://clinicaltrials.gov/ct2/results?recrs=ab&cond=Duchenne+muscular+dystrophy&term=&cntry=&state=&city=&dist=. Accessed 24 November 2018. PMO phosphorodiamidate morpholino oligomer, PPMO peptide-conjugated morpholino oligomer, ENA ethylene-bridged nucleic acids oligonucleotides, ASO antisense oligonucleotides.
